# A novel method for drug-target interaction prediction based on graph transformers model

**DOI:** 10.1186/s12859-022-04812-w

**Published:** 2022-11-03

**Authors:** Hongmei Wang, Fang Guo, Mengyan Du, Guishen Wang, Chen Cao

**Affiliations:** 1 College of Computer Science and Engineering, Changchun University of Technology, Changchun, China; 2 School of Biomedical Engineering and Informatics, Nanjing Medical University, Nanjing, China; 3 Department of Biochemistry and Molecular Biology, Alberta Children’s Hospital Research Institute, University of Calgary, Calgary, Canada

**Keywords:** Drug-target interaction, Graph attention network, Line graph

## Abstract

**Background:**

Drug-target interactions (DTIs) prediction becomes more and more important for accelerating drug research and drug repositioning. Drug-target interaction network is a typical model for DTIs prediction. As many different types of relationships exist between drug and target, drug-target interaction network can be used for modeling drug-target interaction relationship. Recent works on drug-target interaction network are mostly concentrate on drug node or target node and neglecting the relationships between drug-target.

**Results:**

We propose a novel prediction method for modeling the relationship between drug and target independently. Firstly, we use different level relationships of drugs and targets to construct feature of drug-target interaction. Then, we use line graph to model drug-target interaction. After that, we introduce graph transformer network to predict drug-target interaction.

**Conclusions:**

This method introduces a line graph to model the relationship between drug and target. After transforming drug-target interactions from links to nodes, a graph transformer network is used to accomplish the task of predicting drug-target interactions.

## Background

It is well known that there are tens of thousands of diseases that threaten human health. Drug discovery is an important research area that urgently needs to be explored . At the same time, the rapid development of computer technology has sparked a wave of interdisciplinary collaboration. In particular, with the aid of machine learning and deep learning, bioinformatics can effectively improve the efficiency of drug discovery. Drug-target interactions (DTIs) prediction aims to identify the targets of drug molecules, which plays a crucial role in the drug discovery process and has become a hot topic in computer-aided drug discovery [1]. Compared with traditional drug discovery models, DTIs prediction can effectively reduce the cost of drug discovery [2].

Traditional methods of DTIs prediction are mostly based on machine learning. Recent works, such as the fuzzy bipartite local model [3], multi-output prediction method [4], and superior Bayesian personalized ranking method [5] are representative methods. Ding et al. [3] developed a fuzzy bipartite local model based on a fuzzy least squares support vector machine and multicore learning to predict DTIs. They first applied multicore learning to fuse multiple drugs and targets, and finally used fuzzy bipartite local models to infer unknown DTIs. Pliakos et al. [4] proposed DTIs prediction as a multi-output prediction problem and solved it by learning an ensemble of multi-output biclustering trees on a reconfigured network. Ye et al. [5] proposed an Adversarial Bayesian Personalized Ranking model that first generated ternary biased-order relations for drug targets, then used the biased-order relations to train a drug and target latent factor matrix, and finally obtained the score ranking for DTIs prediction from the inner product of latent factors.

With the rapid development of deep learning methods, DTIs methods have been proposed as a deep learning approach for target prediction and drug repurposing in heterogeneous drug-gene-disease networks, which greatly facilitates target identification and advances the process of drug repurposing. Sun et al. [6] proposed an auto-encoder-based DTI prediction method that projects drug features to the protein space via a multi-layer encoder and then to the disease space via a decoder. Xuan et al. [7] proposed methods to integrate multi-scale adjacent topologies, multiple similarities, associations, and drug- and protein-related interactions, which used a fully connected self-encoder learning framework to learn low-dimensional feature representations of nodes in heterogeneous networks, and then applied a multilayer convolutional neural network to generate the final predictions. Howevertraditional methods are frequently used for small samples, and extracting complex graph structure information is difficult.

Since DTIs networks can be modeled as networks, many network based methods have emerged at this stage to predict DTIs. Manoochehri et al. [8] proposed a network topology-based framework for predicting interacting and non-interacting drug-target pairs that is capable of learning complex drug-target topological features. Jin et al. [9] proposed the multi-resolutional collaborative heterogeneous graph convolutional Auto-Encoder method for DTIs prediction, which fused and assigned weights to embeddings of various types of links and continuously added adjacent embeddings by gated recurrent units before fusing them together to form the final embedding. Yue et al. [10] proposed a method for bipartite DTI relations based on heterogeneous network embedding that decomposed a heterogeneous DTI network into three sub-networks. A random forest model was used to predict new DTIs by combining the features of a bipartite DTI network for drug-target interactions, a drug-based similarity network, and a target-based similarity network. However, current deep learning methods are simpler for drug-protein interactions and cannot extract deep-level interaction information.

The existing DTI prediction methods are excellent, but there are still some problems. Researchers normally solely consider drug-protein interactions and overlook drug-protein interactions between two drug-protein pairs. In addition, the relationship between nodes and the whole heterogeneous graph is often neglected. In this paper, we introduce a line graph with drug-protein pairs as vertices and propose a drug-target interaction prediction method based on a graph transformer network (DTI-GTN). The main contributions of our method are as follows.

- Current approaches for DTIs prediction are limited to simple drug-protein interactions. To address this problem, we constructed a drug-protein pair interaction line graph with the drug-protein interactions as vertices, which allows us to extract more information about drug-protein pair interactions.
- Traditional models place more emphasis on the node’s neighbor relationship and less emphasis on the node’s relationship to the whole heterogeneous graph. To solve this problem, we employ the GTN model to determine the relationship between each interaction node and the entire heterogeneous graph.
- Our method contributes to increasing the efficiency of DTIs prediction. The experimental results on the Peng et al. [11] dataset show that our method performs well on both AUROC and AUPR metrics.

The full paper is divided into five parts, which are organized as follows. In background, we introduce the background of the study and presents the main research contents and contributions of this paper in view of some current problems of drug-target interaction prediction and the current status of domestic and international research. There are the related works in the field of drug target prediction and the shortcomings of the current work in Related Works. In methods, we propose a drug-target prediction method based on the GTN model, which transforms the drug-target map into a drug-protein pair line graph and predicts and evaluates it by the GTN model. In addition we describe in detail each module of our method. In experiment, we present the data set used in this paper, the validation metrics and the final results of multiple experiments. After extensive review, it is found that the drug-protein pairs with the highest prediction scores have practical significance, thus confirming the effectiveness of this method. In conclusion, we summarize the entire work and point out the limitations of this study and the outlook for future work.

## Related works

Drug-target interactions (DTIs) prediction plays an important role in finding potential therapeutic compounds. Moreover, DTIs prediction is an indispensable step in drug re-positioning [12] and drug discovery [13]. DTIs prediction is also helpful to identify new ligands for new drugs and targets by identifying the interactions between drug compounds and protein targets. DTIs prediction methods can be roughly divided into traditional methods and deep learning methods. Among a large number of deep learning methods, network-based methods perform well in predicting DTIs. Therefore, the following focuses on the traditional methods and the network-based methods in deep learning.

### Traditional DTIs prediction methods

Traditional DTIs prediction methods are mainly divided into two categories: (1) methods based on molecular docking simulation [14]. (2) Ligand-based approaches [15]. Based on basic biophysical principles and the crystal structure of the target binding site, molecular docking methods often yield good prediction of druggability. In contrast to conventional ligand-protein docking, reverse ligand-protein docking aims to seek potential protein targets by screening an appropriate protein database [16]. Ligand-based approaches are often designed based on the principle of structure-dependent properties. These methods use structural similarity to search similar compounds in terms of activities or treatment mechanisms. Although the above-mentioned methods have shown high prediction accuracy. Those molecular docking methods rely on the three-dimensional structure of the target protein [17]. The results of ligand-based methods may be less than ideal when there are insufficient data on known ligands [18]. However, most current network-based approaches ignore the information relationship between the nodes and the whole heterogeneous graph.

### Network based methods

In recent years, many network-based methods have been proposed to predict potential DTIs because DTIs networks can be modeled as networks. There are some advantages of these methods that do a better use of Network Structure Information [19]. Manoochehri et al. [8] proposed a semi-supervised bipartite graph model. The model integrated drug-drug and protein-protein relationships into a bipartite graph. Jin et al. [9] proposed a multi-resolution collaborative heterogeneous graph convolution autoencoder for DTIs prediction that collaboratively aggregated the learned embeddings from different types of links in heterogeneous drugtarget networks, thus leading to more interpretable embeddings for each drug and target node. Tang et al. [20] proposed a heterogeneous network edge denoising model based on association exponential kernel matrix and potential global association. This method transformed the DTIs prediction problem into a noise reduction problem on heterogeneous networks. The heterogeneous network was constructed by combining drug and target kernel matrices and the existing DTIs network. Furthermore, the method not only used the information of associations of the nearest neighbors to perform DTIs prediction, but also incorporated the global association between drugs and targets to reduce the sparsity of DTIs network and improve prediction accuracy. Yue et al. [10] proposed a heterogeneous network embedding DTIs model, which can extract distinct features from every sub-network of the heterogeneous DTIs network and concatenate these features by the topological information between the sub-networks. This method makes better use of the characteristics of DTIs relationships between both sides and assists similar information and targets related to drugs.

In recent years, graph neural networks have become another hot topic of graph mining. Due to the rapid development of graph machine learning, different graph neural networks have benn proposed [21]. Among them, heterogeneous graph neural network (HGN) [22], hraph attention networks (GAT) [23], Topology adaptive graph convolutional networks (TAG) [24], and residual gated graph convnets (RGG) [25] are representative models [25]. Graphs provide a universal way to represent data, and many other types of data can also be transformed into graphs. Drug side effect prediction and DTIs identification are essentially edge prediction problems. Cheng et al. [26] proposed an end-to-end deep learning approach based on a graph attention network and multiple self-attention mechanisms to predict DTIs. The feature extraction of drugs and proteins is improved by using graph attention network and a multi-head self-attention mechanism. However, they only use one-dimensional data to represent the structural characteristic information of drugs and proteins, and much advanced characteristic information of drugs and proteins is lost in prediction. Peng et al. [11] improved the prediction method by learning low-dimensional vector representations of features from heterogeneous networks, and adopting convolution neural networks (CNN) as classification models. Wang et al. [27] proposed a simple and efficient ligand protein binding prediction model based on a residual graph neural network (GNN) and attention. In this network, the complex graph features are learned through the residual GNN. They integrate these features into the attention module to form a complex protein vector for multilayer perceptron processing. However, most graph neural network-based models only examine the relationships between drugs and proteins and ignore many of the relationships between each group of drugs and proteins. Based on these shortcomings, this paper proposes a graph transformer-based method for predicting DTIs, taking into account the relationship between each group of drug-protein pairs and the information of nodes and the full graph, as a way to predict the interactions between drug targets.

## Methods

**Fig. 1:**
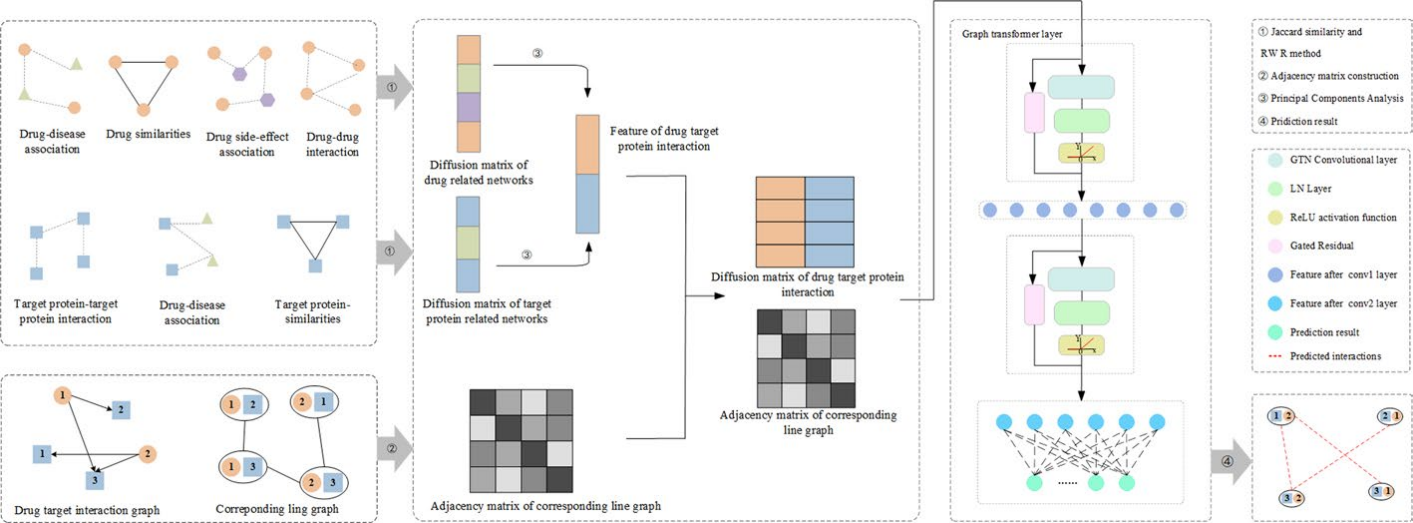
The DTI-GTN method’s flow chart. DTI-GTN includes a feature extractor based on heterogeneous networks, a feature selector based on principal component analysis, a converter to transform drug-protein interaction maps into drug-target pairwise line maps, and a fully connected layer classifier based on the GTN model. First, using the Jaccard similarity coefficient and RWR algorithm, features were retrieved from a network of seven medicines and proteins. Following that, a PCA model is used to reduce the dimensionality of these features, as well as to turn the drug-protein interaction graph into a drug-protein pairwise line graph. Finally, a GTN model was developed to forecast the interactions of each drug-protein pair

We propose a drug-targeted interaction prediction method based on graph transformer network (DTI-GTN). It not only introduces line graphs fusing the relationships between each group of drug-protein pairs, but it also allows GTN models to extract relationships between nodes and the entire heterogeneous graph. Figure 1 depicts the DTI-GTN workflow. We first aggregate multiple drug and protein information sources using Jaccard similarity coefficients to generate similarity matrices for multiple drug and protein networks and then randomly walk the similarity matrices using the restart random walk (RWR) method to generate high-dimensional feature vectors for drugs and proteins. Finally we use principal component analysis (PCA) models to reduce the high-latitude feature vectors of dimers.

The second stage is to create the drug-protein pair interaction line graph. To do so, we first created the drug-protein pairs by selecting the medications and proteins that have an interaction relationship based on the drug-protein adjacency matrix information. Then, using certain guidelines, we generate the edges between the drug-protein pairs as nodes. If the components of two drug-protein pairs have the same drug or protein, the edges between them are formed. Following completion of the preceding process, the interaction line graphs of drug-protein pairs and node features are combined and input into the GTN model so that features can be extracted, and the fully connected layer predicts the association between each two drug-protein pairs to generate prediction results and prediction probabilities.

### Heterogeneous-network-based feature extractor

Heterogeneous networks are constructed based on the following two types of networks. The first type is drug-related networks, including drug-drug interactions, drug-disease associations, drug-side effect associations, and drug similarity (based on the chemical structure of the drug). The other type is the protein-related network, including protein-disease association, protein-protein interaction, and protein similarity (based on the primary sequence of the protein). First, we apply the Jaccard similarity method to each association matrix and interaction matrix to construct a similarity matrix.

In the drug-disease interaction matrix, for example, two rows of the adjacent matrix represent sets A and B, which represent the interactions between two different drugs and all diseases. The Jaccard coefficient of these two sets is the ratio of the size of the intersection of A and B to the size of the concurrent set of A and B. It is a measure of the similarity of two sets. This is how it is defined:

1 $$\begin{aligned} Sim(A,B)=\frac{A\cap B}{A\cup B}. \end{aligned}$$

The Jaccard similarity coefficient is used to compare the similarity and difference between finite sample sets. The greater the value of the Jaccard coefficient, the greater the similarity of the samples. The similarity matrix represents the similarity between each drug or protein node and all features in the column nodes. For example, element $S_{i,j}$ in the original adjacency matrix represents the similarity between row *i* and row *j*.

In the next step, the RWR method [28] is used for each similarity matrix. The basic idea of the random wander method is to traverse a graph starting from a vertex or a series of vertices. At any vertex, the traverser will randomly jump to any vertex in the graph with probability P, which is called the jump occurrence probability. A probability distribution is derived after each tour, which shows the probability that each vertex in the graph will be visited.

The RWR method is an improvement on the random wandering method. The traverser starts from a node in the graph and faces two choices at each step, randomly selecting an adjacent node or returning to the starting node. The RWR method captures the multifaceted relationships between two nodes and the entire graph structure.

According to the RWR principle, the greater the similarity between two nodes, the greater their transfer probability. Thus, if two nodes’ distribution states are similar, they can be considered to be in a similar position with respect to other nodes in the network. This is because the RWR principle states that the greater the similarity between two nodes, the greater the likelihood of a leap between them [29].

Taking the drug-disease similarity matrix $A_{i,j}$ as an example, we can obtain the drug-disease transition transfer matrix *B* based on $A_{i,j}$, where the elements $B_{i,j}$ describe the transition probabilities of drug and disease node *j*, defined as follows:

2 $$\begin{aligned} B_{i,j} = \frac{A_{i,j}}{\sum \nolimits _{j}A_{i,j}}. \end{aligned}$$

Then, the final drug-disease diffusion state matrix is obtained by iterative convergence as follows:

3 $$\begin{aligned} S_i^{t+1}=(1-P_r)S_i^{t}B+p_r e_r \end{aligned}$$

During the random wander, each element stores the probability of entering the disease node after iteration from drug node *i*,$s_i^t$ is the result after *t* is iterated, $p_r$ denotes the probability of restart, and $e_i$ is represented as an n-dimensional unit matrix.

After transforming all similarity matrices into diffusion state matrices, all diffusion state matrices of a drug network and a protein network are stitched together to yield two drug network and protein network diffusion state matrices. The rows of the drug diffusion matrix represent different drugs, and the columns represent the four nodes of drug, disease, side effect, and drug, with the element $d_{i,j}$ representing the probability of transfer between the drug and node j. The protein diffusion state matrix’s rows represent different proteins, and the columns represent protein, disease, and protein nodes, with the element $p_{i,j}$ representing the transfer probability between the protein and node *j*.

### Principal component analysis feature selector

The diffusion state matrix vector obtained in the previous step is high-dimensional, noisy, and incomplete. To obtain the basic features, we manipulate the data using the PCA model [30], and the main processes of the PCA model are shown in the supplementary information.

The goal of PCA is to map high-dimensional data into a low-dimensional space by linear projection, and to maximize the information content of the data in the projected dimension, to use fewer data dimensions while retaining the characteristics of more original data points. Therefore, PCA reduces the dimensionality of the original features while keeping the “information content” as much as possible. In this study, we reduce both drug and protein features to 125 dimensions. In this study, we reduced both drug and protein features to 125 degrees.

### Graph transformer based interaction predictor

The transformer model, introduced by Google in 2017, is still widely used today. This model was first used for machine translation tasks, and it allowed for fast parallelism using the self-attention mechanism. The most criticized drawback of RNNs is slow training, and the transformer model can improve on this drawback. Dwivedi et al. [31] extended the transformer model to graphs to preserve the properties of the graph. Specifically, given the node feature $H^{(l)} = \{ H^{(l)}_1,H^{(l)}_2,\cdots ,H^{(l)}_n \}$, the multi-head attention of each edge from *j* to *i* is calculated as follows.

4 $$\begin{aligned}&q_{c,i}^{(l)} = W_{c,q}^{(l)}h_{i}^{(l)} + b_{c,q}^{(l)} \end{aligned}$$

5 $$\begin{aligned}&k_{c,i}^{(l)} = W_{c,k}^{(l)}h_{j}^{(l)} + b_{c,k}^{(l)} \end{aligned}$$

6 $$\begin{aligned}&e_{c,ij} = W_{c,e}e_{i,j} + b_{c,e} \end{aligned}$$

7 $$\begin{aligned}&\alpha _{c,ij}^{(l)} = \frac{\langle q_{c,i}^{(l)},k_{c,j}^{(l)}+e_{c,ij} \rangle }{\sum \nolimits _{u\in N(i)}\langle q_{c,i}^{(l)},k_{c,u}^{(l)}+e_{c,iu} \rangle } \end{aligned}$$

Among Formula (4) is the exponential scale dot-product function and *d* is the hidden size of each head. For the *C*-th head attention,first transform the source feature and distant feature into $q_{c,i}^{(l)}\in \mathbb {R}^{d}$ and $k_{c,i}^{(l)}\in \mathbb {R}^{d}$ using different trainable parameters $W_{c,q}^{(l)}, W_{c,k}^{(l)},b_{c,q}^{(l)},b_{c,q}^{(l)}$,and then encode the edge features $e_{i,j}$ and add them to the key vector as additional information in each layer.

After obtaining the multi-head attention of the graph, message aggregation is performed for distance *j* to source *i*:

8 $$\begin{aligned}&v_{c,i}^{(l)} = W_{c,v}^{(l)}h_{j}^{(l)} + b_{c,v}^{(l)} \end{aligned}$$

9 $$\begin{aligned}&\hat{h}_{i}^{(l+1)}=\Vert _{c=1}^{C}\left[ \sum _{j\in \mathcal {N}} \alpha _{c,ij}^{(l)}(v_{c,i}^{(l)}+e_{c,ij}) \right] \end{aligned}$$

where *C* is the number of multi-headed attentions, || is the connection to attentions, and $v_c$ is used instead of the distance feature $h_j,j\in \mathbb {R}^{d}$ for weighted sum.

Furthermore, according to Shi et al. [32]. Use a multi-headed attention matrix instead of the original normalized adjacency matrix as the transfer matrix for message passing, use a gated residual connection between layers to prevent the model from being too smooth, and finally apply graph transformer on the final output layer to apply averaging on the multi-headed output and remove the non-linear transformation.

## Experiment

### Dataset

We evaluated the performance of the DTI-GTN method using a drug-target interaction prediction task.

We obtain the dataset from Peng’s paper [11], which contains 12,015 nodes and 1,895,445 edges. In this dataset all isolated nodes are excluded. This heterogeneous network integrates four types of nodes (drug, protein, disease and side effect) and six types of edges (drug-protein interaction, drug-drug interaction, drug-disease association, drug-side effect association, protein-disease association and protein-protein interaction). Peng et al. also extract information from known DTIs and drug-drug interactions based on multiple databases to extract multiple information, drug nodes from the DrugBank database [33] and protein nodes and protein interactions from the Human Protein Reference Database [34]. Disease nodes, drug-disease and protein-disease associations were extracted from the Comparative Toxicogenomics Database [35]. Side effect nodes and drug side effect associations were obtained from the side effect resource [36].

First, we create some drug-related and protein-related similarity matrices. Drug-related similarity matrices include the drug-drug similarity matrix, drug-disease similarity matrix, drug-side effect similarity matrix and drug similarity matrix. Protein-related similarity matrices include the protein-disease similarity matrix, protein-protein similarity matrix and protein similarity matrix.

We next use the RWR algorithm to stitch together the diffusion state matrices of the drug and protein networks, resulting in two diffusion state matrices representing the drug and protein, respectively. The rows of the drug diffusion matrix represent different drugs, the columns represent proteins, diseases, side effects and drug nodes, and the values in the matrix represent the associations between the drugs and the four biological entities. The rows of the protein diffusion state matrix represent the different proteins. The columns indicate protein, disease, and drug nodes, and the values in the matrix show the associations between the proteins and the three biological entities. We next used the PCA model to downscale the drug diffusion state matrix and protein diffusion state matrix, yielding 708 drug feature vector matrices with 125 dimensions and 1512 protein feature vector matrices with 125 dimensions, respectively.

In the next step, we construct the line graph. First, the drug and protein nodes with the presence of edges are used as a new pair of drug-protein pair nodes according to the drug-protein interaction relationship, so that each pair contains information about the drug and the protein. Next, the edges of the line graph are constructed based on the relationship between each group of drug-protein pairs, and a new adjacency matrix representing the relationship between the drug-protein pair nodes is obtained. Finally, we obtain the new drug-protein pair node features based on splicing the 125-dimensional drug features with the 125-dimensional protein features.

Following completion of the preceding steps, the training and test sets were divided, with 80% of the positive and negative samples used as the training set, 10% of the positive and negative samples used as the validation set, and 10% of the positive and negative samples used as the test set. The known drug-protein interaction pairs were used as positive samples based on the known drug-protein interaction matrix, with a total of 40,058 positive samples, and the same number of negative samples as positive samples were randomly selected. The final experimental results were calculated as the mean plus or minus the standard deviation of the five training predictions, ensuring that the experimental results were accurate.

### Parameters of models

**Fig. 2:**
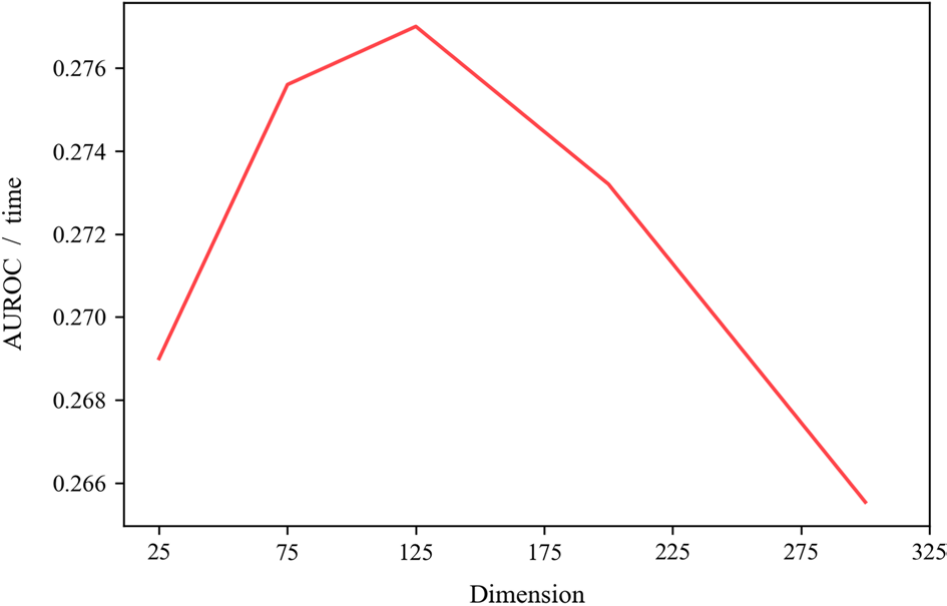
Values of different dimensional data are compared using AUROC/time. Whereas higher AUROC values and shorter time periods indicate better model performance

For the RWR model, according to the parameters of the Peng et al. [11] model, we restart with a probability of 0.5 and a number of 20 iterations. Our original drug feature input dimension is 2832, and our protein feature input dimension is 4536, and we use the PCA model to reduce dimensionality. The dimensionality is chosen as shown in Fig. 2, and the value of AUROC/time varies with dimensionality, with 125 dimensions providing the best balance. The final dimension was set to 125. The GTN model was run for 2000 batches and optimized with the Adam method at an initial learning rate of 0.001, with the loss calculated as a cross-entropy loss.

### Evaluation metrics

Model testing and comparison are performed using AUROC [37] and AUPR [38] scores, which are commonly used evaluation criteria for machine learning and represent the area under the ROC curve and PR curve, respectively. The higher the score is, the higher the prediction accuracy of the model and the better the performance of the model.

The ROC curve is a curve with the probability of false positives (FPR) as the horizontal axis and the probability of true positives (TPR) as the vertical axis.

10 $$\begin{aligned} FPR=\frac{FP}{FN+FP}. \end{aligned}$$

11 $$\begin{aligned} TPR=\frac{TP}{TP+FN}. \end{aligned}$$

Using the classification gives the probability of a positive class for each instance. Then, by setting a threshold value such as 0.6, a probability greater than or equal to 0.6 is considered a positive class, and a probability less than 0.6 is considered a negative class. The corresponding set of (FPR, TPR) can be calculated, and the coordinate points in the plane can be obtained. As the threshold value decreases, an increasing number of instances are classified as positive classes, but these positive classes are also mixed with true negative instances, i.e., TPR and FPR will both increase. The coordinate point (0,0) corresponds to the maximum threshold value, and the coordinate point (1,1) corresponds to the minimum threshold value. The ROC curves are depicted in the Supplementary Material.

THE PR curve is a curve with recall as the horizontal axis and precision as the vertical axis.

12 $$\begin{aligned} Recall=\frac{TP}{TP+FN}. \end{aligned}$$

13 $$\begin{aligned} Precision=\frac{TP}{TP+FP}. \end{aligned}$$

The PR curves still reflect the classification performance well in the case of large differences in positive and negative sample proportions, as shown in the AUPR schematic in the Supplementary Information.

### Baselines

The DTIs prediction task can be viewed as a binary classification problem, where known drug-protein pair interactions can be considered positive samples and unknown drug-protein pair interactions can be considered negative samples. In the experimental procedure, all positive samples were collected first, and then the number of positive samples was used as an example to randomly sample the negative samples. Next, 80% of the positive and negative sample pairs in the dataset were randomly selected as the training set to train the model parameters, 10% of the data were used as the validation set to adjust the hyperparameters of the model and for initial evaluation of the model capabilities, and finally the remaining 10% of the data were used as the test set to evaluate the generalization ability of the final model. In our experiments we compared DTI-GTN with six state-of-the-art graph neural network methods. Including (1) SSCGCN: Instead of using Laplacian Matrix to convolve the graph, this model uses Chebyshev polynomials as the convolution kernel, and the larggest feature is that it does not need to decompose the feature vector. (2) GAT: The shortcomings of previous problems such as graph-based convolution are addressed by using masked self-attentive layers. By sacking layers (in which nodes are able to aggregate the features of their neighbors), different weights can be assigned to different nodes in the neighborhood without any expensive matrix operations or prior knowledge of the graph structure. (3) GCN : proposes a scalable semi-supervised learning method for graph structure data, which is based on an efficient variant of convolutional neural networks that can directly manipulate graphs. (4) EGC : uses a new adaptive filtering method that achieves lower memory consumption and latency and is suitable for gas pedal implementation. (5) Hypergraph: introduces hypergraph convolution and hypergraph attention in the family of graph neural networks. Hypergraph convolution defines the basic formula for performing convolution on hypergraphs, while hypergraph attention further enhances representation learning by utilizing the attention module. (6) ResGatedGraphConv: The LSTM and ConvNets models for graphs are proposed, iterating over the graph multiple times and introducing the idea of residual networks to enable the model to scale to graphs of arbitrary size. (7) In GNN-FiLM, the representation of the target node of an edge is used to compute a transformation that can be applied to all incoming messages, allowing featurewise modulation of the passed information.

### Performance evaluation on predicting drug-target interactions

To ensure the accuracy of the experimental results and avoid pseudo-random results, all models are trained five times under the same conditions, with the results averaged and standard deviations added and subtracted. The final AUROC and AUPR values for each model are shown in Table 1. The AUROC value of DTI-GTN is 0.9973, which is 0.0017 higher than that of the next best model DTI-Film. The AUPR value is 0.0018 higher than that of DTI-Film. In the drug-target interaction prediction task, DTI-GTN outperformed the other six state-of-the-art DTIs prediction methods.

**Table 1:** Comparison with graphical neural network models based on line graphs

Model	AUROC	AUPR
DTI-GTN	0.9973 ± 0.0006	0.9976 ± 0.0006
DTI-Film	0.9956 ± 0.0007	0.9958 ± 0.0008
DTI-GAT	0.9946 ± 0.0004	0.9948 ± 0.0002
DTI-GCN	0.9935 ± 0.0006	0.9942 ± 0.0003
DTI-EGC	0.9928 ± 0.0010	0.9945 ± 0.0003
DTI-RGG	0.9811 ± 0.0028	0.9844 ± 0.0022
DTI-Hypergraph	0.9796 ± 0.0059	0.9829 ± 0.0045

**Fig. 3:**
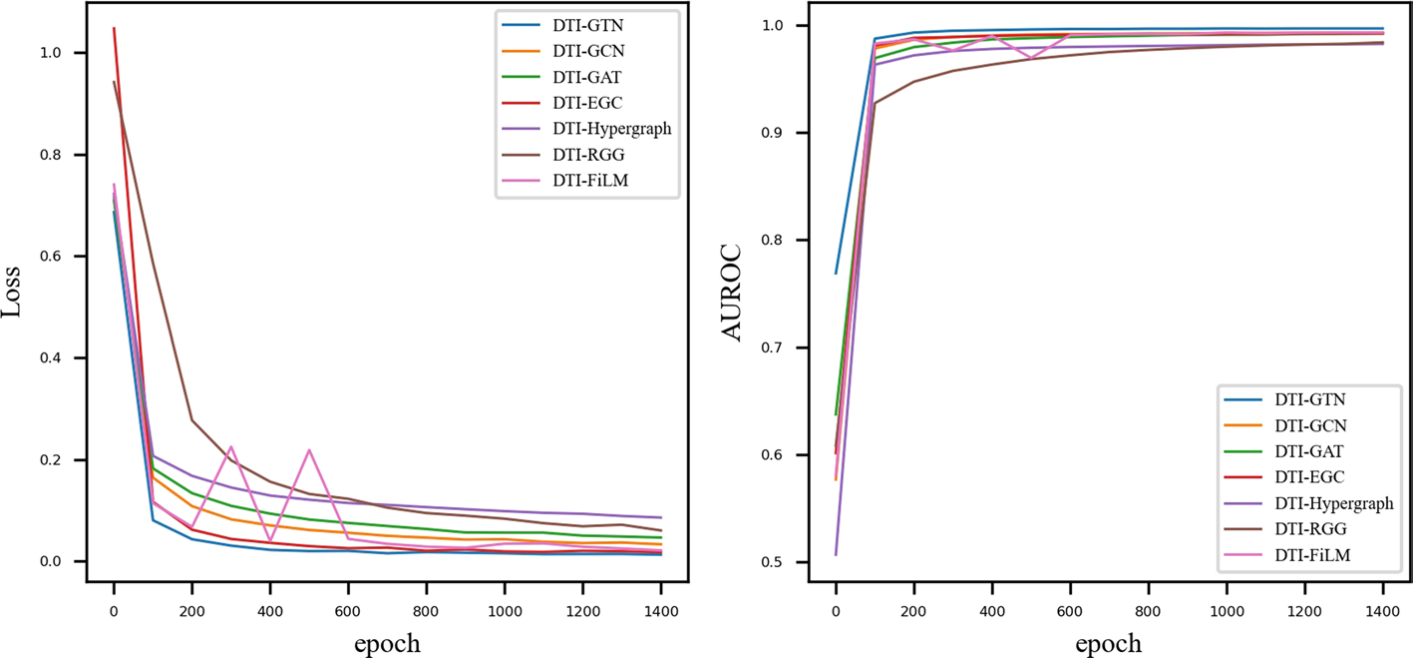
ROC (**a**) and P-R (**b**) curves of DTI-GTN, DTI-GCN, DTI-GAT, DTI-EGC, DTI-HGC, DTI-RGG, and DTI-FiLM in the prediction tasks for drug-target interactions. With an AUOC of 0.9958 and AUPR of 0.9969, DTI-CNN performed better in the prediction tasks for drug-target interactions, possessing better results than other methods

Meanwhile, Fig. 3 depicts the trends in training loss and ROC values for various methods during the training process. According to the two figures, the training loss of all seven models gradually decreases and the ROC value gradually increases as the epoch value increases, but when compared, their convergence speed differs. The DTI-GTN method, which is faster and better than the other models, begins to converge after approximately 200 rounds.

In addition, we also compare DTI-GTN with other models and its classical learning methods. (1) DTI-CDF: In this method the prediction performance of DTIS is further improved by using path classification-based multi-similar features of DTIs heterogeneous graphs and a depth-cascaded deep forest-based model (CDF). (2) DTI-CNN: In this method a self-coding model with restarted random wandering and denoising is used to handle incomplete, high-dimensional heterogeneous features of the data source. A deep cnn model is used to process low-dimensional feature vectors and predict the probability of interaction between each pair of drugs and proteins. (3) Random forest: In this method for each node, m features are randomly selected and the decision of each node in the decision tree is determined based on these features. Based on these m features, the best way to split them is calculated so that each tree is constructed. (4): K nearest neighbors: In this method given the training dataset, for a new input instance, find the K instances that are closest to the instance, then the new input instance belongs to the same class as the majority of these K instances.

**Table 2:** Comparison of Ablation Study results of DTI-GTN method

Model	AUROC	AUPR
DTI-GTN	0.9973 ± 0.0006	0.9976 ± 0.0006
DTI-CDF	0.9898 ± 0.0019	0.9901 ± 0.0005
DTI-CNN	0.9903 ± 0.0015	0.9918 ± 0.0016
DTI-RF	0.9488 ± 0.0024	0.9708 ± 0.0020
DTI-KNN	0.8769 ± 0.0030	0.9057 ± 0.0038

Similarly, the experimental results were averaged over five trials plus or minus the standard deviation, and the final results are shown in Table 2. DTI-CDF performed the best among the other classical methods, but GTI-GTN outperformed it by 0.0075 and 0.0075 for AUROC and AUPR, respectively, when compared to the other four classical methods, and DTI-GTN also performed the best in the drug-target interaction prediction task.

Table 3 compares the prediction results of our DTI-GTN method to those of other models that do not use line graphs, and in this experiment, we add two representative graph neural network models for comparison. (1) NEDTP [39]: This method uses 15 heterogeneous information networks to build a similarity network, and after extracting topological information using random wandering, the gradient boosting decision tree model is used to complete the classification task. (2) Moltrans [40]: This method uses a knowledge inspired sub-structural pattern mining algorithm and an augmented transformer encoder to capture the relationships between substructures for a more accurate prediction of DTI interactions. The AUROC and AUPR values for the model without using the line graph in Table 3 are lower than those of the model using the line graph in Tables 1 and 2, demonstrating the effectiveness of our use of the line graph.The AUROC and AUPR values in Table 3 for the model without the line graph are lower than those in Tables 1 and 2, demonstrating the effectiveness of our use of the line graph.

**Table 3:** Comparison of baseline models based on interaction diagrams of DTIs

Model	AUROC	AUPR
DTI-GTN	0.9973 ± 0.0006	0.9976 ± 0.0006
DTI-Film	0.8512 ± 0.0300	0.8925 ± 0.0182
DTI-GAT	0.8802 ± 0.0087	0.9179 ± 0.0038
DTI-GCN	0.8771 ± 0.0095	0.9267 ± 0.0310
DTI-EGC	0.8679 ± 0.0184	0.9005 ± 0.0082
DTI-RGG	0.8544 ± 0.0139	0.9004 ± 0.0120
DTI-Hypergraph	0.8353 ± 0.0087	0.8839 ± 0.0167
NEDTP	0.9355 ± 0.0049	0.9428 ± 0.0118
Moltrans	0.8596 ± 0.0063	0.8608 ± 0.0079
DTI-CDF	0.8689 ± 0.0112	0.9037 ± 0.0096
DTI-CNN	0.9341 ± 0.0017	0.9417 ± 0.0136
DTI-RF	0.8640 ± 0.0088	0.9035 ± 0.0081
DTI-KNN	0.7727 ± 0.0204	0.8320 ± 0.0127

**Fig. 4:**
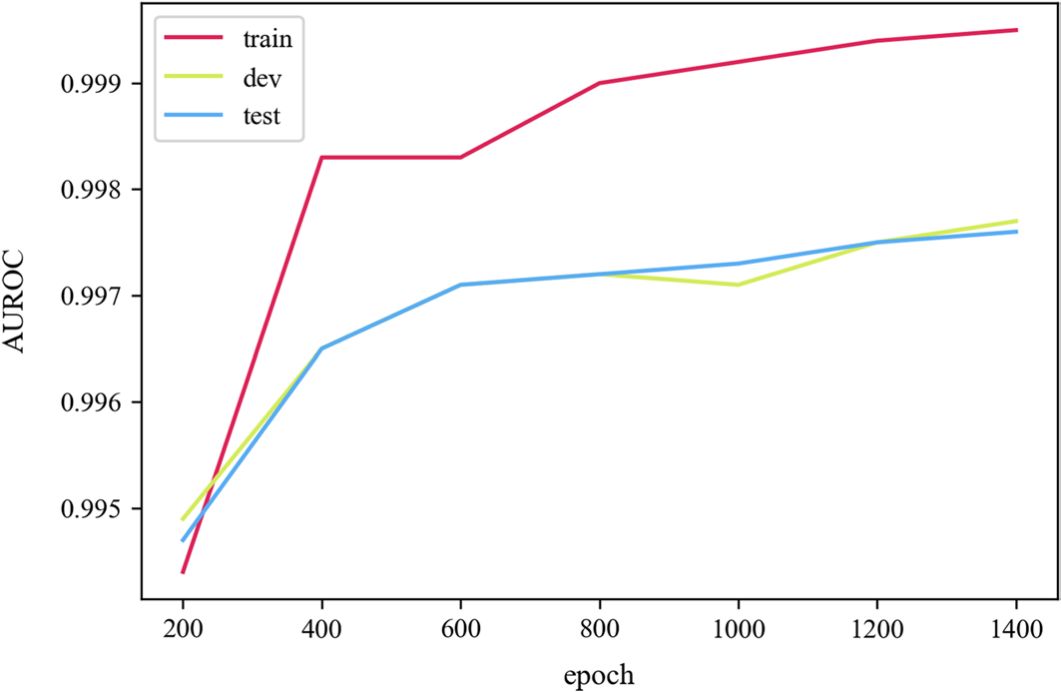
AUROC comparison of the training set, validation set and test set during model training

Figure 4 depicts the AUROC change curves of the training, validation, and test sets during model training. The figure shows that the AUROC of the test set, which is not used in training at all, and the AUROC of the validation set are roughly equal, indicating that model training has good generalization ability and there is no risk of overfitting.

### Ablation study

The ablation experiments of our model are shown in Table 4, with the GTN module removed and the line graph removed. As shown in the table, the mean AUROC and mean AUPR of the model decreased by 0.0237 and 0.0300 with the GTN module removed, and by 0.1147 and 0.0812 with this part of the transformed line graph removed. This demonstrates the utility of our GTN model and the line graph conversion module.

**Table 4:** Comparison of AUROC and AUPR values for ablation experiments

Model	AUROC	AUPR
DTI-GTN	0.9973 ± 0.0006	0.9976 ± 0.0006
Without GTN module	0.9760 ± 0.0037	0.9676 ± 0.0240
Without line graph module	0.8826 ± 0.0064	0.9164 ± 0.0042

### Case study

We divided the dataset into a training set and a test set. Predictions were made for all drug target pairs in the test set. We selected three pairs of drug-protein pairs with the top 3 prediction scores from the model prediction results for validation, and the results and scores are shown in Table 5. Each drug-protein pair includeed two drug-protein interactions. The prediction results for the three groups of drug-protein pairs are shown in Table 5. Each set of drug-protein pairs corresponds to two predicted results. For example, if A and C are drugs, B and D are proteins, and AB and CD are a set of predicted drug-target pairs, it is demonstrated that drug A interacts with protein D and drug C interacts with protein B. Our prediction results were checked in Drugbank, and the test results were further analyzed.

**Table 5:** AUROC, AUPR values for drug-target interaction prediction tasks

	DRUG ID	DRUG	PROTEIN ID	PROTEIN	Prediction score
1	DB00960	Pindolol	P08908	HTR1A	22.1942
1	DB00571	Propranolol	P08908	HTR1A	22.1942
2	DB00315	Zolmitriptan	P08908	HTR1A	19.1483
2	DB00952	Naratriptan	P08908	HTR1A	19.1483
3	DB01226	Mivacurium	P20309	CHRM3	18.3155
3	DB01337	Pancuronium	P20309	CHRM3	18.3155

5-Hydroxytryptamine receptor 1A is abbreviated as HTR1A. The two pairs of drug-protein pairs with the highest predicted results were Propranolol-HTR1A and Pindolol-HTR1A. Propranolol [41] has a significant affinity for HTR1A. Pindolol [42] is a beta adrenoceptor antagonist. It facilitates frontocortical dopaminergic and adrenergic transmission primarily by activation of beta 1/2-ARs and, to a lesser degree, by stimulationing HTR1A receptors. In addition, the selective HTR1A receptor antagonist can slightly attenuate the pindolol-induced increase in DA and NAD levels.

second set of drug-protein pairs in the prediction results are Zolmitriptan-HTR1A and Naratriptan-HTR1A. Zolmitriptan [42] is a novel 5-hydroxytryptamine receptor agonist with proven efficacy in the acute treatment of migraine with or without preceding aura. Naratriptan [43] has a central effect in the trigeminovascular system, selectively inhibiting afferent activity in cardiovascular neurones, via HTR1B, HTR1D and HTR1A receptors.

Muscarinic acetylcholine receptor M3 is abbreviated as CHRM3. The last pair of drug-protein pairs are Mivacurium-CHRM3 and Pancuronium-CHRM3. Mivacurium [44] is a short-acting non-depolarizing neuromuscular blocking agent. Muscle relaxants cause bronchospasm via histamine release or by acting on muscarinic receptors. Pancuronium [45] is a neuromuscular blocker used as an adjunct to general anesthesia to facilitate tracheal intubation. Neuromuscular blocking drugs can inhibit not only nicotinic but also muscarinic receptors and thereby affect not only skeletal but also smooth muscle tone.

## Conclusion

We propose a novel drug-target prediction model based on graph transformer network (DTI-GTN) in this paper. Firstly, we use seven different level relationships of drugs and targets to construct features of drug-target interaction with jaccard similarity and random walk with restart method. Then, we use line graph to transform drug-target interaction from nodes into links of a new graph. After that, we introduce graph transformer network to predict drug-target interaction. We compare our model with other representative models on AUROC and AUPR values. The experiment results on DTIs network show our model is comparable with other models. Our DTI-GTN method can provide a new pattern for understanding drug-target interaction relationship.

## Data Availability

The dataset and code used in the current study are available at the github repository [https://github.com/q498756498/DTI-GTN].

## Abbreviations

DTI: Drug-target interactions
AUROC: The area under the receiver operating characteristics curve
AUPR: The area under the Precision and Recall curve
TPR: True positive rate
FPR: False positive rate
PCA: Principal component analysis
GTN: Graph transformer network
RWR: Random walk with restart
